# Economy in Motor Tyres

**Published:** 1909-05-01

**Authors:** 


### Motoring Notes.

### ECONOMY IN MOTOR TYRES.

Messrs. Harvey, Frost and Co., the inventors of the well-known process of tyre repairing, some time ago forwarded me an advance copy of their book, "Economy in Motor Tyres." This has been published with the view of assisting the motorist in his choice of tyres, and to give some hints on their care and treatment. By the co-operation of all the principal tyre companies the author has been able to give a large amount of official information, which I am sure will prove of considerable service to motorists of all classes.

Messrs. Harvey, Frost inform me that they will be pleased to forward a copy of this useful volume post free, to anyone applying for same to their offices at 39 Great Eastern Street, E.C. I would strongly advise readers of this journal to write for a copy of this little book, which contains a vast amount of concise yet comprehensive information. No motorist can afford to remain indifferent to the question of tyre economy. Not only are the tyres the most important and expensive items in the equipment of a car, but their upkeep is of equal importance from a pecuniary standpoint. It is therefore imperative that the car-owner who desires to reduce his expenses to the lowest possible figure should give this matter his closest attention.

### The Choice of Tyres.

With regard to the choice of tyres there are many valuable hints contained in the various lists of the tyre manufacturers, but the difficulty has been that, as in the nature of things the manufacturers are competitive, these hints and details have hitherto been spread over a large number of lists, which have to be gathered together if the motorist wishes to examine the claims and peculiarities of each tyre. And even then he may omit, or be ignorant of, the very list which contains particulars of the tyre which would best suit his purpose. For a long time past some means has been required whereby the whole official and authoritative data could be collected in one publication and presented to the public by a disinterested and impartial method. This object has now been gained by the issue of this book by Messrs. Harvey, Frost.

It requires but a cursory glance at the articles contributed by the various motor-car tyre companies to see that the life of the tyre depends on its care and treatment, and that the manufacturers are unanimous in this respect. The mere fact that the same make of tyres on the same class of car vary in behaviour according to the different drivers in charge proves conclusively that in many instances the drivers are themselves to blame when tyre troubles arise. The manufacturers all emphasise the importance of proper inflation. Pumping too hard increases the wear and lessens the resiliency of the covers, in addition to reducing the comfort of the occupants of the car. If not hard enough, then the canvas becomes chafed and worn through friction, and the rubber is also more liable to cut near the rims.

Careful driving, at a reasonable speed, the avoidance of sharp turns and of sudden, hard use of the brake unless absolutely necessary, are all points to be observed if the driver desires to treat his tyres well. When in the garage the tyres should be properly protected from contact with oil, grease, and paraffin in any form whatever; kept away from bright sunshine, and not allowed to remain standing in pools and puddles of water. In short, they should be treated in a common-sense manner, and the care expended will be amply repaid by the prolonged life, the decreasing amount of repair bills, and economy in time, temper, and purse.

### Maxims for the Care of Tyres.

The little book I allude to above gives an excellent summary of points to be attended to in regard to tyres, some of which I reproduce here:—

Inspect the tyres after every long run and attend to cuts at once.

Repair them in a proper manner and avoid all makeshift methods.

Fit the heaviest tyres your car can take.

Use the best quality tubes; poor quality is the reverse of economy.

When fitting a tyre or tube don't fail to use sufficient French chalk; a lubricant between cover and tube is absolutely necessary.

Take care that tyres do not stand in oil and grease in the garage.

Don't fail to use protectors for spare tyres carried on the car.

Don't abuse the use of clutch and brake.

Don't forget to carry spare valve parts.

Don't neglect to use a good pressure-gauge.

Don't damage tyres by taking sharp turns.

Don't wait until your tyres are in a bad way; attend to them immediately any signs of damage appear.

Don't use damaged rims, sometimes caused by running on deflated tyres.

The foregoing rules are all most excellent, and, if carefully followed, will undoubtedly considerably lessen the expense of tyre upkeep, nowadays the heaviest item to be considered in regard to motor-car maintenance. Many experienced motorists, however, whilst attending to the other points, habitually neglect the last rule in regard to examining their rims from time to time. This is really necessary, since every rim which is rusted or flattened soon produces cuts round the beads of the cover. The French for "bead" is *talon* or "heel," and as Achilles, though otherwise invulnerable, was killed through a wound in the heel, so many a cover has been done to death by a wound in the bead.

"Viator."

